# Is the patient satisfaction questionnaire an acceptable tool for use in a hospice inpatient setting? A pilot study

**DOI:** 10.1186/1472-684X-13-27

**Published:** 2014-06-02

**Authors:** Kate ME Henriksen, Naomi Heller, Anne M Finucane, David Oxenham

**Affiliations:** 1 Marie Curie Hospice Edinburgh, Frogston Road West, Edinburgh EH10 7DR, UK; 2 College of Medicine and Veterinary Medicine, University of Edinburgh, 49 Little France Crescent, Edinburgh EH16 4SB, UK

**Keywords:** Patient satisfaction questionnaire, Hospice, Specialist palliative care, Revalidation, Multisource 360° feedback

## Abstract

**Background:**

The Patient Satisfaction Questionnaire (PSQ) is an assessment tool used to evaluate patients’ perspectives of their doctor’s communication and interpersonal skills. The present pilot study investigated whether the PSQ could be administered successfully in a hospice inpatient setting and if it is an acceptable tool for completion by patients and relatives in this context.

**Methods:**

The study was conducted in two phases. A first phase was undertaken to establish the process of PSQ administration in a hospice inpatient ward. A second phase of questionnaire administration followed by semi-structured interviews explored inpatient experiences of the questionnaire process.

**Results:**

Overall, 30 inpatients and one relative were invited to complete the PSQ across both phases of data collection, representing 53% of all inpatients at the time of data collection. The remaining 47% were deemed unsuitable to ask due to a diagnosis of dying (24%), confusion (17%), distress (3%) or lack of availability (2%). The average response rate across both phases of data collection was 87%. Qualitative interview data suggested that the PSQ was considered clear, easy to understand and not burdensome in terms of time or effort for this population.

**Conclusions:**

The PSQ appears an acceptable tool to use in a hospice inpatient setting. Many patients welcomed the opportunity to be involved and give feedback. Using a greater proportion of relatives as an alternative source of feedback could be considered in future studies.

## Background

The Patient Satisfaction Questionnaire (PSQ) is an assessment tool used to evaluate patients’ perspectives of their doctor’s communication and interpersonal skills. It is used to inform the continued professional development of medical staff in the UK. The General Medical Council’s (GMC) guidance, “Ready for Revalidation”, [1] suggests that all doctors with patient contact will be expected to complete a PSQ at least once per five year revalidation cycle, both as a learning and development tool and to inform the revalidation decision.

A number of PSQs have been developed to evaluate patients’ perspectives of their doctor’s communication and interpersonal skills. None has as yet been prescribed for the revalidation process. Studies on the use of both the GMC questionnaire [2] and the Joint Royal Colleges of Physicians Training Board (JRCPTB) questionnaire [3] report that patient responses are stable over time, show good internal consistency and correlate well with other measures of performance [4,5]. The GMC deems PSQs appropriate for use provided they are consistent with the principles, values and responsibilities set out in the GMC’s core guidance of Good Medical Practice [6]. A minimum number of completed forms are recommended to ensure robust reliability (16 to 20 for the JRCPTB [5,7], 20 according to the Royal College of Physicians [8] and 34 for the GMC questionnaire [4,9]).

Good communication is at the heart of palliative care practice and the concept of PSQ feedback is central to maintaining standards; however, there are a number of challenges. Hospice settings are very different to the high volume outpatient clinic environment which lends itself to the PSQ process. The nature of the hospice inpatient population, with most patients in the terminal stage of their illness, limits the number of patients well enough to participate, potentially necessitating lengthy time-scales for sufficient data collection. Clinicians may also have concerns that patients under their care might feel pressurised to complete a questionnaire [10,11].

Some authors have suggested that patients approaching end of life issues are too vulnerable to participate in research or service improvement [11]. However, others believe that while palliative care users face particular difficulties, these should not be advanced as reason not to provide equal opportunities for those who want to be involved [12]. More recently research has shown that many people using palliative care services are “*capable of deciding whether to participate in interviews and negotiating* [interview] *circumstances*” [13].

The first phase of this pilot study explores if it is feasible to administer a PSQ effectively in a hospice inpatient unit; while the second phase explores whether hospice inpatients and relatives consider the PSQ an acceptable way to collect feedback.

## Methods

### Design

Two phases of data collection were conducted as part of this pilot study. During the first phase all eligible patients were invited to complete a PSQ. During the second phase, all eligible patients were invited to complete a PSQ followed by a short semi-structured interview.

### Setting/Context

Both phases of the study were undertaken within an inpatient unit at Marie Curie Hospice Edinburgh. The ward used during the first phase of data collection had 14 beds. During the second phase the hospice had temporarily relocated and data collection occurred in one large inpatient ward of 22 beds.

### Patient Satisfaction Questionnaire

The PSQ used for this study was adapted from that suggested by the JRCPTB [3]. This is a 12 item questionnaire examining patients’ perspectives of their doctor’s behaviour and effectiveness in a consultation, focusing on professionalism, interpersonal and communication skills (Additional file 1).

### Eligibility criteria

The lead ward nurse, having collated information from the wider multidisciplinary team decided which patients should be invited to complete a PSQ. All inpatients were considered eligible unless they were dying; were judged to be confused; or judged to be experiencing a high level of psychological distress. The doctor undergoing the PSQ process was not involved in deciding which patients were eligible. Patients were only eligible to participate once in the PSQ process for an individual doctor.

### Procedure

#### ***Phase 1***

The PSQ, together with an explanatory letter (Additional file 2) was given to each eligible patient by the ward clerk following the doctor’s ward round once a week over a seven week period. If appropriate, a relative was provided with a questionnaire on behalf of the patient (e.g. where an eligible patient was unavailable, but a relative who had attended the consultation was available). Administrative staff were available to assist patients with the process if requested, and collected the completed questionnaires. To ensure confidentiality and anonymity, patients posted their completed questionnaires into a sealed box. No patient identifiable data was recorded on the questionnaire. All questionnaires related to patient experience of a single doctor.

#### ***Phase 2***

PSQs were administered in the same way as during Phase 1. Subsequent to administering the PSQ, patients who had completed a questionnaire were invited to participate in a recorded, semi-structured interview to explore their experiences of the questionnaire process, their interest in participation, and any feelings of coercion. All participants were interviewed in the hospice inpatient unit. Interviews were conducted by the second author (NH), a fourth-year medical student at the University of Edinburgh. Participants were provided with a participant information sheet (Additional file 3) and participant consent was recorded verbally and in a written form prior to the interview taking place. As the second phase of the study was designed to assess the acceptability of the PSQ process, a compacted phase of data collection was undertaken over a two week period by two doctors to generate a sufficient number of interviews for qualitative data collection.

### Quantitative data collection and analysis

For both phases of the study, data was collected relating to the number of patients and relatives approached to participate in the PSQ process. Data was also collected on the number of patients not approached and the reasons for this. This data provided an indication of the proportion of hospice inpatients that could be asked to complete a PSQ over a given period of time and the length of data collection that may be required to achieve sufficient PSQ responses to ensure robust reliability as per JRCPTB guidance.

### Qualitative data collection and analysis

Ten semi-structured interviews were conducted in phase 2 (9 patients and 1 relative). The interview guide is shown in Additional file 4. Data was digitally recorded and transcribed by the interviewer (NH). Interview data was thematically analysed by the interviewer and two other members of the study team (KH and AF).

### Ethics and governance arrangements

Given this study constituted an evaluation of service, the South East Scotland Research Ethics Committee advised that formal NHS ethical approval was not required. Approval was given by the hospice Caldicott Guardian.

## Results

### Quantitative findings

During the first phase of data collection 21 of 36 inpatients were invited to complete the PSQ. Of the 15 patients not approached, this was due either to confusion, a diagnosis of dying or distress as determined by the multidisciplinary team. Seventeen completed questionnaires were returned, equating to an 81% response rate.

**Figure 1:**
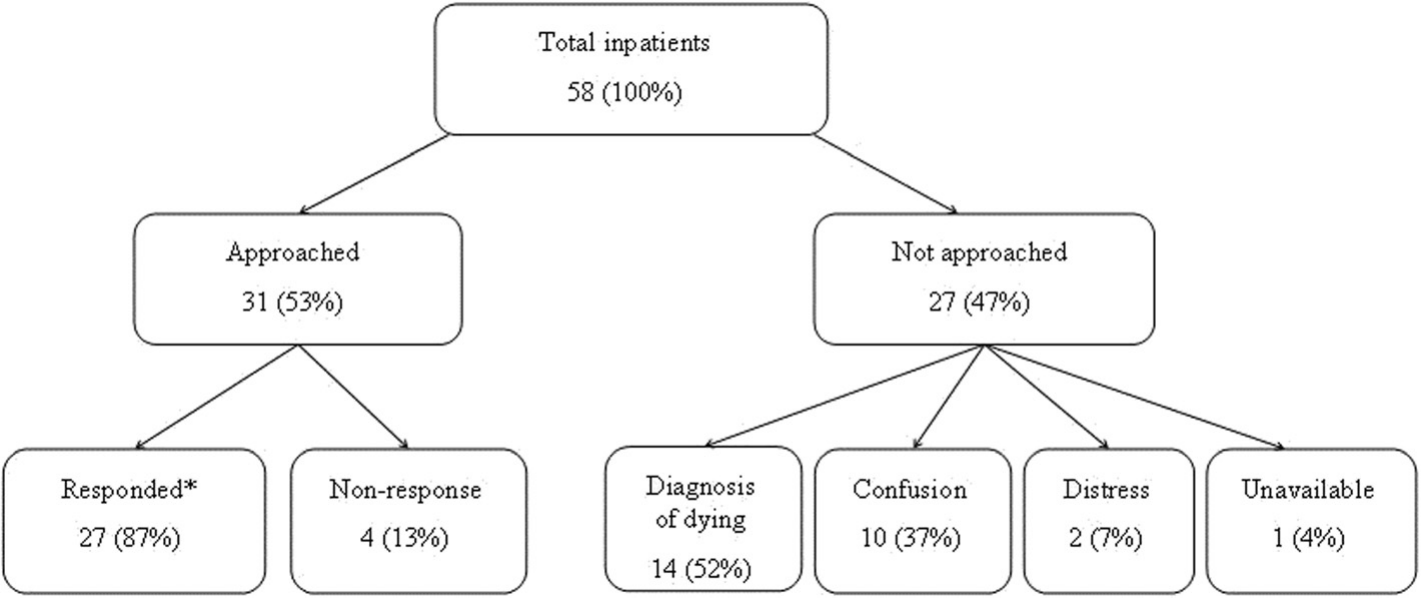
Number of patients completing the PSQ based on combined data from phase 1 and 2.

During the second phase of data collection, 9 patients and 1 relative were invited to take part in both the PSQ and subsequent interview process. All agreed to participate. Twelve of a total of 22 inpatients were not approached due to confusion, dying, distress, or being unavailable at the time.The combined data from both phases of data collection revealed that 53% of inpatients at the time of PSQ administration were deemed suitable to approach for PSQ completion. The vast majority of those approached completed the questionnaire. Of those not approached, the main reasons were that the patient was dying or confused (Figure 1).

### Qualitative findings

Interview findings suggested that patients found the PSQ clear, easy to understand, and not burdensome in terms of time or effort. They also recognised the value of a collective voice and some viewed the process as an opportunity to be involved and empowered. The key findings are described below (*P* is used to denote the participant, *I* denotes the Interviewer).

Patients reported that they found the PSQ clear and easy to understand:

*“very clear questions, no problem” (P3) “she actually read them out for me….I thought was better*” (P10)

The PSQ was not perceived to be burdensome in terms of time or effort:

“you want it to be simple and it was” (P1) “it was very easy for me to fill in” (P3) “effort-if you’re a bit sleepy-but no it was ok” (P2)

Participants felt they could be honest in their PSQ responses:

“I was perfectly honest…no point filling it in if you’re not” (P4) “because it’s anonymous so its not going to come back to you” (P2) “it went in an anonymous envelope and my name wasn’t on it” (P2)

Some participants considered themselves a valuable resource for information and feedback while a number of participants recognised their contribution had potential to benefit the doctor:

“I think it’s good-if they are doing a good job they get to know” (P2) “if there are things that they could change then maybe (it) give(s) them a chance to think” (P2) “it’s actually to help the doctor” (P1)

Some participants recognised the value of a collective voice:

“if all the patients are saying the same thing maybe this would highlight something” (P2) “if a core of people are saying something..” (P4)

Some participants felt it rational for their opinion to shape service improvement:

*“how else do people get information, unless they ask questions” (P8)* “*it’s the only way you’re going to find out what’s what*” (P4)

A number of participants viewed the PSQ as an opportunity to be involved and empowered:

“you feel more involved in what is going to happen to you” (P3) “I like to give an opinion in the hope of being heard… you always hope” (P4) “my answers all meant something” (P5)

All ten interviewees, when asked directly, said they would have felt able to decline participating in the PSQ process had they wished. However, two participants said that some people may find it difficult to decline:

“it’s kind of a bit hard to say no …you would probably just want to fill it in” (P2)

None of the ten participants explicitly stated that they had any concerns that their responses might influence their subsequent care:

*I: “ Do you think that people might have any concerns that their answers might affect their care in any way?” P1*: *“I don’t think so no, because it says that it is just to find out you know how they got on with the doctor really”.*

The interview data suggested that there may be potential for PSQ responses to be biased by external circumstances, or by how the patient feels about the general hospice environment or care they are receiving, as opposed to communication with their doctor:

“the care is fantastic…they are lovely everybody…it is lovely to be able to have flowers on the ward as well” (P3)“I was getting home then all of a sudden…you’re not going home…you just want to go home” (P7)

Occasionally it was not clear that the participant fully understood what was being asked of them, although all participants were deemed capable of completing the PSQ by the multidisciplinary team. One participant was not clear which member of the clinical team she had been speaking with prior to being asked to complete the survey:

“was that a doctor…” (P10)

## Discussion

### Principal findings

Measuring patient/user satisfaction will be a requirement for all UK medical practitioners as a means of informing continued professional development and revalidation. This study demonstrates that administering a PSQ in a hospice inpatient setting is both feasible and acceptable to patients. Furthermore, the questionnaire is not burdensome to participants who are deemed well enough to approach from the outset. Most reported that they did not feel obliged to respond, and none admitted concerns that their responses may influence subsequent care. This study also found that approximately half of all patients in a hospice inpatient setting were deemed suitable to approach with a PSQ; the main reasons not to approach patients related to dying and confusion.

### Fit with current literature

Some authors have argued that patients dealing with end-of-life issues are too vulnerable to participate in research or service improvement [10,11]. Consequently potentially interested patients are often prevented from participating as a result of gate-keeping by concerned members of staff and/or families [14,15]. The findings from our interviews resonate with the alternate view that the decision to participate in research and related studies is often motivated by the opportunity to make a contribution, and to be ‘of help’ to other people [16,17]. Furthermore, reflecting the view that there are positive personal impacts associated with involvement [13,18] our findings suggest that patients receiving specialist palliative care also value the opportunity to provide feedback and to have the chance to make a difference.

A GMC survey on PSQ implementation suggested that the PSQ process is potentially open to reporting bias due to a number of factors such as patient age, ethnicity and mechanism of questionnaire return [4]. Our interview analysis also suggests a potential for bias depending on how the patient was feeling at the time they were approached for PSQ completion (e.g. physical environment, feelings of comfort, general care, expectation about discharge). It is likely that such bias can be minimised by clearly communicating with patients that the questionnaire relates specifically to their experience with their doctor, and not to their general experiences about their care. Ensuring that the recommended number of completed PSQs is generated will also increase reliability of the results.

There are challenges to surveying this patient population as approximately half of all patients in an inpatient setting may be unable to complete a questionnaire due to physical or psychological limitations. Furthermore, even when a patient is deemed able to participate in the PSQ process by the multidisciplinary team, they may not fully understand what is being asked of them. This is unlikely to be unique to palliative care - care of the elderly, paediatrics, psychiatry, learning disability and many other fields may be similarly affected. In such contexts, it is particularly important that the staff member responsible for inviting the patient to take part, takes a moment to assess the patient’s understanding of what is required of them, and gives them the opportunity to ask questions. If the patient is deemed to not fully understand what is being asked, other sources of feedback such as relatives should be considered**.** Interestingly, we did not generate significant numbers of completed PSQs from relatives, which may have been due to the post ward round timing of the questionnaire process. The feasibility and usefulness of approaching relatives where the patient is unable to take part in the PSQ process could be explored in future studies.

### Study strengths and limitations

This study is the first to examine the PSQ process in a hospice inpatient setting. It provides valuable information for those required or wishing to seek feedback from such patient populations using PSQ tools. The views expressed in the interviews here represent about half of all patients using a palliative care inpatient service at a given period in time. Patients who were dying, confused or experiencing psychological distress were excluded based on the judgement that responses from these patients may be inaccurate or that compassionate care for the individual was in conflict with requesting their involvement. Data collection exploring the reasons why patients were not invited to complete a questionnaire was a strength of this study. It is recommended that multidisciplinary teams are explicit about the reasons why patients are deemed inappropriate to approach, to ensure that bias is reduced where possible.

A further strength of the study was that the interviewer (NH) was not a member of the clinical team caring for the patients she was interviewing. This is hoped to have facilitated open and honest responding. However, it is impossible to guarantee that all participants were completely open and honest in their responses. Some may have had concerns that they didn’t actually express. Highlighting that the patient questionnaire was anonymous, and could not be traced back to an individual patient was important to a number of our participants and likely to be key in promoting unbiased responding.

This was a pilot study focused on PSQ administration in one hospice inpatient unit. The results are not generalizable to community or other such settings for which other studies are recommended.

### Implications for clinical practice

The GMC recommends that PSQs should be administered in a sequential and unselected fashion. However, in a specialist palliative care inpatient unit, about half of all inpatients may be too unwell to ask at a given point in time. Furthermore patients may spend several days in the inpatient unit, limiting the number of new patients that can be approached from one day to the next. These constraints will extend the time required to gather sufficient numbers of patient questionnaires. In phase 1 of this pilot study, it took seven weeks for one doctor to collect 17 questionnaires in a 14 bed hospice inpatient unit. The period required for data collection will depend on ward size and patient length of stay so is likely to vary across local contexts.

Two of the 10 interviewees in the second phase of the study required additional help to complete the questionnaire. Appropriate administrative support clearly needs consideration when putting a PSQ process into practice and is crucial to its success.

Highlighting the anonymity of all patient data collected is important. In this study a number of patients commented specifically on the importance of anonymity and were particularly reassured by the PSQ supporting documentation emphasising this together with the visible measures taken to ensure this (i.e. sealed box for return of anonymous questionnaires).

This pilot study focused specifically on the feasibility and acceptability of administering a PSQ tool in a palliative care inpatient setting. There are a number of PSQ tools available to doctors, with different guidance relating to administration and the minimum number of questionnaires required for a valid response. It is important that doctors using a PSQ, use the appropriate guidance relating to the tool they choose to use [1,7-9]. Further work on the validity and reliability of PSQs is recommended, especially given the time involved in administering the questionnaires in settings such as palliative care inpatient units.

## Conclusion

The PSQ appears to be an acceptable tool to use in a hospice inpatient setting. The intended purpose of the PSQ and reassurance as to anonymity must be clearly communicated to the patient to facilitate participation. Establishing explicit criteria for participation is recommended to find the appropriate balance between protecting patients who are deemed too unwell to take part whilst minimising gate-keeping or self-selection on the part of the clinical team or doctor involved. Obtaining sufficient numbers of completed questionnaires to ensure reliability will remain a challenge and we would encourage consideration of alternative sources of feedback, such as relatives present during the consultation, where patients are unable to give feedback directly. This pilot study provides good evidence for the potential of PSQs to provide feedback for doctors, and to benefit and empower participants in palliative care inpatient setting.

## Competing interests

The authors declare that they have no competing interests.

## Authors’ contributions

KH and DO conceived of the study. All authors contributed to study design. AF analysed the quantitative data. NH collected the qualitative data. KH, NH and AF analysed the qualitative data. KH drafted the paper. All authors read, approved and contributed to the final paper.

## Pre-publication history

The pre-publication history for this paper can be accessed here:

http://www.biomedcentral.com/1472-684X/13/27/prepub

## Supplementary Material

Additional file 1 Patient satisfaction questionnaire. Click here for file

Additional file 2 Cover letter circulated to patients or family member alongside questionnaire. Click here for file

Additional file 3 Participant information sheet. Click here for file

Additional file 4 Question guide used by interviewer. Click here for file
