# Supplementary material for: Is the patient satisfaction questionnaire an acceptable tool for use in a hospice inpatient setting? A pilot study
Source: BMC Palliat Care. 2014 Jun 2;13:27. doi: 10.1186/1472-684X-13-27 (PMC4066835; doi:10.1186/1472-684X-13-27)
Supplement: Additional file 1 — Patient satisfaction questionnaire. [file 1472-684X-13-27-S1.doc]

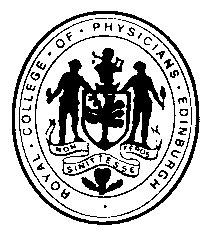


**What did you think of this doctor?**

|  | ***Yes, definitely*** | ***Yes, to some extent*** | ***Not***  ***really*** | ***Definitely not*** | ***Does not apply*** |
| --- | --- | --- | --- | --- | --- |
| Was the doctor polite and considerate? | **🞏** | **🞏** | **🞏** | **🞏** | **🞏** |
| Did the doctor listen to what you had to say? | **🞏** | **🞏** | **🞏** | **🞏** | **🞏** |
| Did the doctor give you enough opportunity to ask questions? | **🞏** | **🞏** | **🞏** | **🞏** | **🞏** |
| Did the doctor answer all your questions? | **🞏** | **🞏** | **🞏** | **🞏** | **🞏** |
| Did the doctor explain things in a way you could understand? | **🞏** | **🞏** | **🞏** | **🞏** | **🞏** |
| Are you involved as much as you want to be in the decisions about your care and treatment? | **🞏** | **🞏** | **🞏** | **🞏** | **🞏** |
| Did you have confidence in your doctor? | **🞏** | **🞏** | **🞏** | **🞏** | **🞏** |
| Did the doctor respect your views? | **🞏** | **🞏** | **🞏** | **🞏** | **🞏** |
| If the doctor examined you did he or  she:   1. Ask your permission? | **🞏** | **🞏** | **🞏** | **🞏** | **🞏** |
| 1. Respect your privacy and dignity? | **🞏** | **🞏** | **🞏** | **🞏** | **🞏** |
| By the end of the consultation did you feel better able to understand and/or manage your condition and your care? | **🞏** | **🞏** | **🞏** | **🞏** | **🞏** |

**Overall, how satisfied were you with the doctor you saw?**

❒ Very satisfied ❒ Fairly satisfied ❒ Not really satisfied ❒ Not at all satisfied

**Please make any additional comments about the doctor in the space below**

**Please remember that this is just about the doctor you have seen today**

**About you -The patient**

**Your gender** ❒ Male ❒ Female

**Your age** ❒ Under 16 ❒ 16 - 30 ❒ 31 - 45 ❒ 46 - 60 ❒ 61 - 75 ❒ 76+

**Who is filling out this form?**

❒ You - the patient ❒ Family member or carer ❒ Facilitator ❒ Interpreter

**Is English your first language?** ❒Yes ❒ No **Today’s date**: _________
