# Supplementary material for: Is the patient satisfaction questionnaire an acceptable tool for use in a hospice inpatient setting? A pilot study
Source: BMC Palliat Care. 2014 Jun 2;13:27. doi: 10.1186/1472-684X-13-27 (PMC4066835; doi:10.1186/1472-684X-13-27)
Supplement: Additional file 3 — Participant information sheet. [file 1472-684X-13-27-S3.docx]

**Additional file 3: Participant information sheet**

**
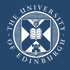
** **
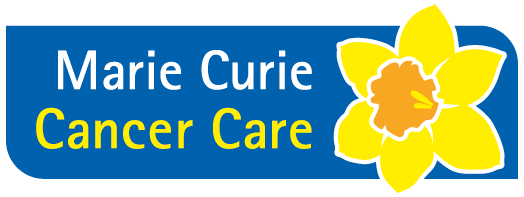
**

Patient Information

Earlier today or this week you participated in a patient satisfaction survey and answered some questions about what you thought of your doctor. The survey was titled: **“What did you think of this doctor”**. We are interested to find out how you and others feel about being asked to participate in this survey, which evaluates doctors’ communication skills.

We are asking for between 10-30 minutes of your time to ask you a few questions after you have completed the survey.

Your interviewer will be [Researcher Name], who is not part of your medical care team.

With your consent, the interviews will be recorded digitally but no information will be recorded that can identify you at any stage. After the interviews have been transcribed and analysed, they will all be destroyed. You are free to withdraw at any time in the process.

The information we collect from the interview will help us understand how patients and caregivers feel about completing surveys about their doctors.

Your participation would be greatly appreciated, if you have any questions please feel free to ask at any time.

Many thanks,

[Researcher Name] [Researcher email address]
