# Supplementary material for: Is the patient satisfaction questionnaire an acceptable tool for use in a hospice inpatient setting? A pilot study
Source: BMC Palliat Care. 2014 Jun 2;13:27. doi: 10.1186/1472-684X-13-27 (PMC4066835; doi:10.1186/1472-684X-13-27)
Supplement: Additional file 4 — Question guide used by interviewer. [file 1472-684X-13-27-S4.docx]

**Additional file 4: Question guide used by interviewer**

*Note: The interview guide was used in a flexible manner. While the questions tended to be asked in the same manner, there was some variation across interviews to enhance the flow of the interview.*

1. How did you feel about being approached to complete a patient satisfaction survey about your interaction with the doctor?
2. Was it clear what you were being asked to do?
   1. If not, why not?
3. Could you tell me what you think is positive about completing this sort of survey, if anything?
4. What about the negative features? Was it burdensome in terms of time and effort?
5. Is there anything else that you feel about the process?
6. Was there anything that you didn’t understand or would’ve liked more explanation about
   1. Was there anything that you needed help with?
7. We are interested to know if patients feel as though they can be really honest in their feedback – were you able to express yourself honestly in your responses, do you think?
8. Do you think that people would have any concerns that their answers may affect their care in any way?
   1. Did this influence you at all?
9. Was there anything about the process that you would like to have changed?
10. Are there any other comments you would like to make?
11. Do you have any questions for me?

At a later stage in the interviews some people were also asked “If your communication with the doctor today had not been as you had hoped, do you feel this was a good way to feed back?”
