# Supplementary material for: Is the patient satisfaction questionnaire an acceptable tool for use in a hospice inpatient setting? A pilot study
Source: BMC Palliat Care. 2014 Jun 2;13:27. doi: 10.1186/1472-684X-13-27 (PMC4066835; doi:10.1186/1472-684X-13-27)
Supplement: Additional file 2 — Cover letter circulated to patients or family member alongside questionnaire. [file 1472-684X-13-27-S2.docx]

**Additional file 2: Cover letter circulated to patients or family member alongside questionnaire**

What did you think of this Doctor?

Dear Patient

To help improve the care your doctor provides, we would like you to complete this questionnaire. In doing so, please comment on the way the Doctor behaved today. Please do **not** comment about other members of staff (such as nurses, receptionists or other doctors in the team).

**THIS QUESTIONNAIRE IS ONLY ABOUT THE DOCTOR YOU HAVE SEEN**

**TODAY.**

Everything you write will be kept confidential and will not compromise your ongoing care in any way. Please be frank when you fill in the questionnaire and feel free to write any comments you wish about the doctor.

You do not have to fill in this questionnaire if you do not want to.

If you are filling this in for a patient please ask them the questions and put their answers (not yours) on the form.

Please fill in the questionnaire after seeing the doctor and our ward clerkess will collect it from you.

Thank you for taking the time to fill in this questionnaire, and if you wish to discuss this further please let us know and we will be happy to do so.

[Name of Medical Director]

Medical Director
